# Microbial diversity and anaerobic hydrocarbon degradation potential in an oil-contaminated mangrove sediment

**DOI:** 10.1186/1471-2180-12-186

**Published:** 2012-08-30

**Authors:** Luiza L Andrade, Deborah CA Leite, Edir M Ferreira, Lívia Q Ferreira, Geraldo R Paula, Michael J Maguire, Casey RJ Hubert, Raquel S Peixoto, Regina MCP Domingues, Alexandre S Rosado

**Affiliations:** 1 Laboratório de Ecologia Molecular Microbiana, Instituto de Microbiologia Paulo de Góes, Universidade Federal do Rio de Janeiro, Rio de Janeiro, Brazil; 2 Laboratório de Biologia de Anaeróbios, Instituto de Microbiologia Paulo de Góes, Universidade Federal do Rio de Janeiro, Rio de Janeiro, Brazil; 3 School of Civil Engineering and Geosciences, Newcastle University, Newcastle upon Tyne, UK

**Keywords:** Mangrove, Bacterial diversity, Anoxic sediment, Sulphate, Petroleum, Hydrocarbons

## Abstract

**Background:**

Mangrove forests are coastal wetlands that provide vital ecosystem services and serve as barriers against natural disasters like tsunamis, hurricanes and tropical storms. Mangroves harbour a large diversity of organisms, including microorganisms with important roles in nutrient cycling and availability. Due to tidal influence, mangroves are sites where crude oil from spills farther away can accumulate. The relationship between mangrove bacterial diversity and oil degradation in mangrove sediments remains poorly understood.

**Results:**

Mangrove sediment was sampled from 0–5, 15–20 and 35–40 cm depth intervals from the Suruí River mangrove (Rio de Janeiro, Brazil), which has a history of oil contamination. DGGE fingerprinting for *bamA*, *dsr* and 16S rRNA encoding fragment genes, and qPCR analysis using *dsr* and 16S rRNA gene fragment revealed differences with sediment depth.

**Conclusions:**

Analysis of bacterial 16S rRNA gene diversity revealed changes with depth. DGGE for *bamA* and *dsr* genes shows that the anaerobic hydrocarbon-degrading community profile also changed between 5 and 15 cm depth, and is similar in the two deeper sediments, indicating that below 15 cm the anaerobic hydrocarbon-degrading community appears to be well established and homogeneous in this mangrove sediment. qPCR analysis revealed differences with sediment depth, with general bacterial abundance in the top layer (0–5 cm) being greater than in both deeper sediment layers (15–20 and 35–40 cm), which were similar to each other.

## Background

The Deepwater Horizon oil spill of 2010 in Gulf of Mexico serves as a reminder of the potential adverse impacts of petroleum compounds to the environment [1,2]. Petroleum is a complex mixture of saturated and aromatic hydrocarbons, polar compounds, resins and asphaltenes. Saturates are proportionally the most significant fraction by mass while the most toxic and persistent compounds are the polar and aromatic hydrocarbons [3]. Such compounds can be responsible for massive wildlife death soon after oil spills and, as well as over the medium and long-term [1]. Unfortunately, accidents resulting in oil spills happen routinely, and due to tidal activity spilled oil is commonly transported to coastal regions. In temperate to tropical latitudes mangrove forests are one of the ecosystems that are most detrimentally affected [4].

Mangroves are vital ecosystems for coastal protection. Their features make them a unique environment, with high biological diversity and activity. Salinity and organic matter availability vary in different parts of mangrove forests [5]. Beneath a thin aerobic surface layer, mangrove sediments are predominantly anaerobic, i.e., anaerobic biochemical processes are catalyzed by sediment microbial communities [6]. In previous studies about microbial populations, it was shown that *Alphaproteobacteria* dominated the bacterial community in a non-disturbed Brazilian mangrove sediment [5] and that after crude oil exposure, bacterial groups such as *Anaerolinea* decrease in population abundance whereas *Deltaproteobacteria* increase [7]. The anoxic nature of mangrove sediment is a key feature that allows oil accumulation in such ecosystems [8]. For example, after an oil spill it is possible to detect higher amounts of oil in deeper sediment than at the surface, showing that oil tends to percolate through the sediment down to deeper layers [9,10].

Several microorganisms are capable of degrading aliphatic and aromatic hydrocarbons under anoxic conditions [11]. Boopathy [12] studied diesel degradation in estuarine sediment microcosms in the presence of different terminal electron acceptors. In the presence of nitrate, sulphate and carbonate, 99% of the crude oil was removed within 510 days, whereas stimulating only sulphate reduction, methanogenesis, or nitrate reduction resulted in 62, 43, and 40% oil removal, respectively. Boopathy and colleagues observed the same interesting results on anaerobic oil hydrocarbon degradation in follow-up studies, showing that sulphate-reducing condition is the most efficient redox condition in experiments using individual electron acceptors [13,14].

Petroleum hydrocarbon degradation pathways are distinct. It is believed that n-alkane-utilizing strains do not grow with aromatic hydrocarbons, and vice versa [15]. There are two elucidated mechanisms for anaerobic alkane degradation. One involves fumarate addition to the alkane subterminal carbon to produce alkylsuccinate compounds, and in the other process the alkane is carboxylated [16]. The enzymes responsible for fumarate addition in anaerobic alkane metabolism are alkylsuccinate synthases, AssA1 and AssA2, encoded by *assA1* and *assA2* genes, respectively [17,18].

Aromatic hydrocarbons are converted to a few central intermediates before being further metabolized. The most common central intermediate of the anaerobic aromatic hydrocarbon transformation is benzoyl-CoA [19], which is then converted to dienoyl-CoA. The next set of reactions ends with a 6-OCH-hydrolase enzyme opening the aromatic ring of the compound. This enzyme is encoded by *bamA* which is considered as a good genetic marker for studying anaerobic aromatic hydrocarbon degradation, since it contains highly conserved regions [20]. However, *bamA* is involved in anaerobic aromatic hydrocarbon degradation in general, and not exclusively degradation of petroleum-derived compounds. Another good target for the detection of anaerobic aromatic hydrocarbon-degrading microorganisms is the enzyme benzylsuccinate synthase (Bss), which is involved in the anaerobic degradation of toluene and xylene, via fumarate addition to the methyl group, transforming these compounds into benzylsuccinates. Bss has been identified in all anaerobic toluene-degrading microorganisms studied to date, and is composed by three subunits, of which, α subunit, encoded by *bssA* gene is the target for molecular studies. This gene is highly conserved and has been employed as a molecular marker for the characterization of environmental samples [20-22].

Despite the importance of crude oil pollution in coastal environments, little attention has been paid to bacterial diversity and anaerobic degradation potential of crude oil hydrocarbons in mangrove sediments. Therefore, the aims of this study were: to compare microbial community profiles in sediments from different depths; to quantify total bacteria and sulphate-reducing bacteria (SRB) as a function of depth; and to screen for the presence of key genes involved in anaerobic hydrocarbon degradation in mangrove sediment.

## Results

### Sediment porewater sulphate concentration

In the current study, sulphate was measured at each studied depth, and in the surface sediment (0–5 cm layer), its concentration was 14.9 mM. Sediment from the two other studied depths, 15–20 cm and 35–40 cm, had a sulphate concentration of 3.6 mM. This suggests an active sulphate reduction zone in the top 15 cm of the sediment. These values reflect the influence of seawater (28 mM sulfate) in mangrove ecosystems, which is introduced by tidal activity.

### Sediment microbial community analyses: PCR-DGGE for 16S rRNA, *bamA* and *dsr* genes

**Figure 1:**
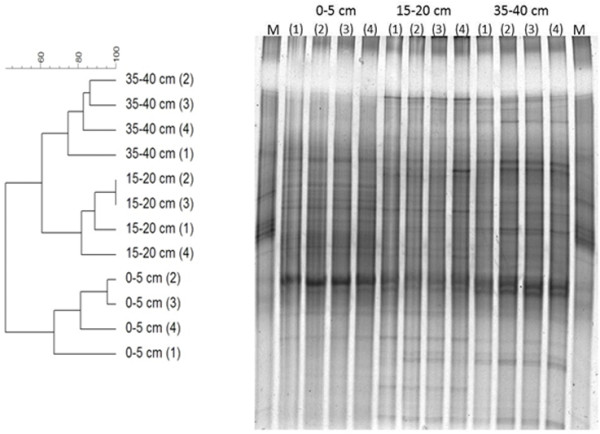
**16S rRNA dendrogram for different depths of mangrove sediment and the gel image.** Dendrogram generated based on denaturing gradient gel electrophoresis (DGGE) fingerprints of 16S rRNA gene fragments from triplicates of mangrove sediment from 3 different depths: 0–5, 15–20 and 35-40 cm, and the DGGE gel image.

To study the bacterial community profile, genomic DNA extracted from sediment samples was analysed by PCR using universal primers to amplify 16S rRNA gene fragments. Amplicons with the expected size of 430 kb were separated by denaturing gradient gel electrophoresis (DGGE) and the results showed a clear distribution of the bacterial populations within the three studied depths (Figure 1), revealing the occurrence of two main clusters: one cluster from the 0–5 cm layer, and another associated with sediment samples from both 15–20 and 35–40 cm depth.

**Figure 2:**
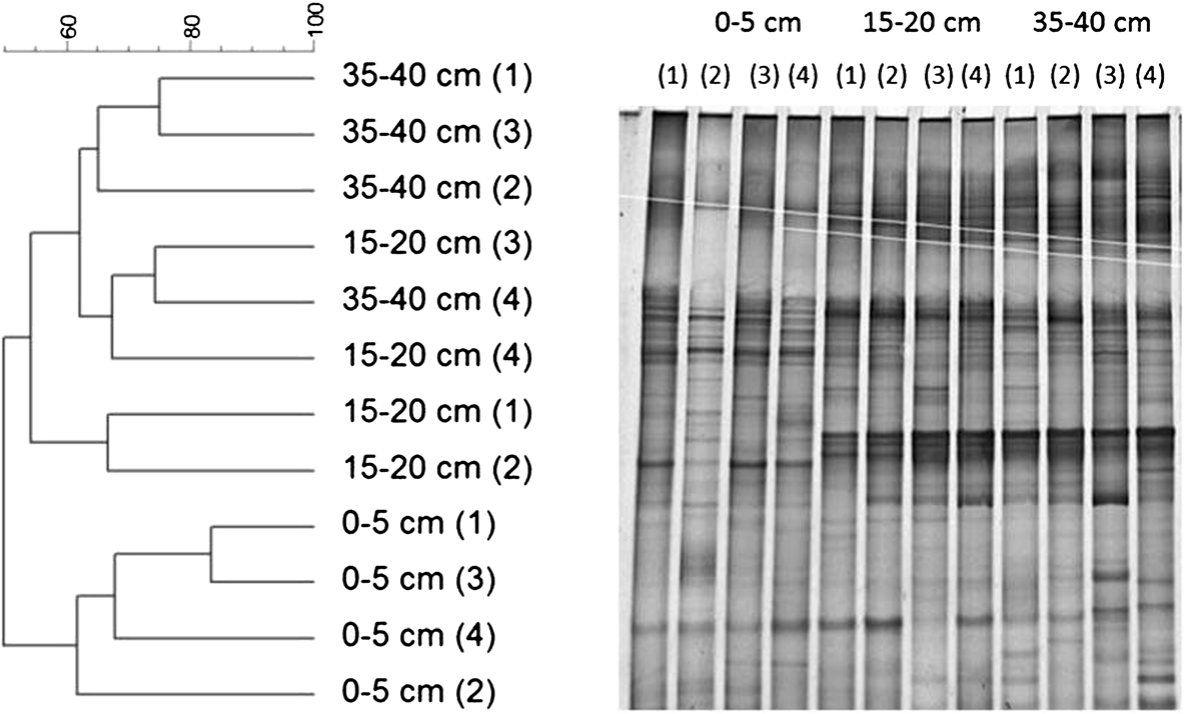
***dsr *gene dendrogram and gel image for different depths of mangrove sediment.** Dendrogram generated based on denaturing gradient gel electrophoresis (DGGE) fingerprints of *dsr* gene from triplicates of mangrove sediment from 3 different depths: 0–5, 15–20 and 35-40 cm, and the gel image.

To study the SRB community at different sediment depths PCR-DGGE was performed using primers targeting the *dsr* gene that encodes the dissimilatory bi-sulphite reductase enzyme that is present in all sulphate reducers [23]. This revealed the occurrence of two main clusters, one cluster derived from the 0–5 cm sediment and the other was associated with sediment samples from both 15–20 and 35–40 cm depth (Figure 2).

**Figure 3:**
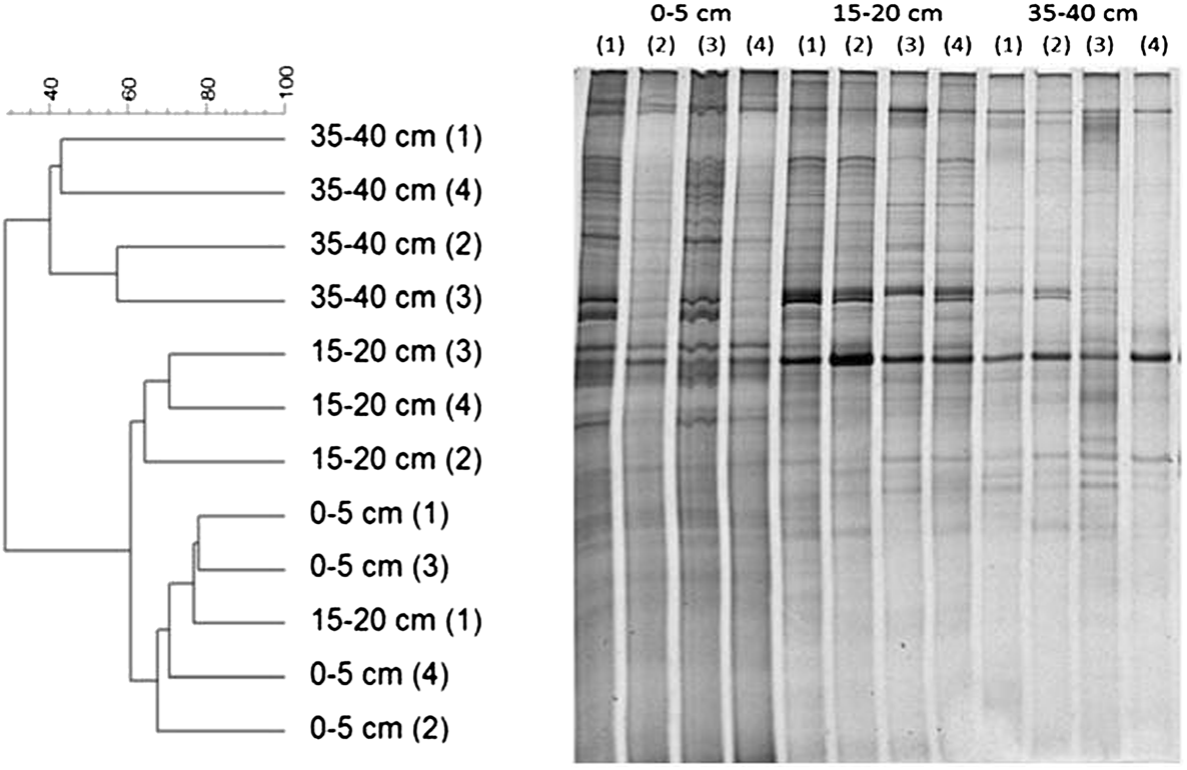
***bamA *gene dendrogram and gel image for different depths of mangrove sediment.** Dendrogram generated based on denaturing gradient gel electrophoresis (DGGE) fingerprints of *bamA* gene from triplicates of mangrove sediment from 3 different depths: 0–5, 15–20 and 35-40 cm, and the gel image.

PCR-DGGE using primers targeting the *bamA* gene, responsible for anaerobic aromatic hydrocarbon degradation, revealed a distribution of two main clusters. Unlike the 16S rRNA gene and *dsrAB* patterns, *bamA* distributions were revealed by one distinct banding pattern common to both the 0–5 and 15–20 cm depths, and a different pattern in the deeper 35–40 cm sediment (Figure 3). The patterns in the shallower sediments can further be clustered specifically to the 0–5 and 15–20 cm sediment depths.

### Molecular techniques for sediment: PCR for *assA* and *bssA*

To further verify the potential for anaerobic petroleum hydrocarbon degradation within the sediment microbial populations, end-point PCR analyses targeting *assA* and *bssA* genes were performed. Genomic DNA from all three sediment depths did not give rise to a PCR product using these primers, despite the fact that this mangrove sediment has a history of petroleum contamination.

### Molecular techniques for sediment: q-PCR for 16S rRNA and *dsr* genes

**Figure 4:**
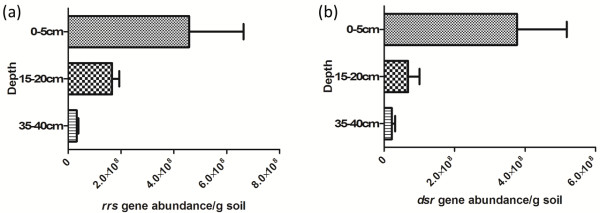
**Bacterial abundance at different depths of mangrove sediment.** Abundance of bacterial populations on mangrove sediments of three different depths tested with q-PCR using oligonucleotide primers for 16S rRNA gene encoding fragment **(a)** and oligonucleotide primers for *dsr* gene **(b)**. Bars with the same letter are not significantly different (one-way ANOVA).

To estimate the bacterial abundance within the three depth horizons, a quantitative (q-) PCR assay was performed for 16S rRNA genes using sediment genomic DNA samples as templates. Results presented in Figure 4a show depth variations of total bacterial 16S rRNA genes. In the top sediment, q-PCR detected 4.6 × 10^8^ genes/g of sediment, in the middle layer, 1.78 × 10^8^ genes/g of sediment, and in the deep sediment, the abundance was 3.2 × 10^7^ genes/g of sediment. One-way ANOVA indicated that the only significant difference was detected between the 0–5 and the 35–40 cm layers.

In order to estimate the abundance of SRB in the sediment samples, q-PCR was performed for *dsr*. The results were used to compare SRB abundance as a function of sediment depth and are shown in Figure 4b. In the top sediment, q-PCR detected 3.6 × 10^8^ genes/g of sediment, in the middle layer 6.6 × 10^7^ genes/g of sediment were detected, and in the deeper layer the abundance was 2.1 × 10^7^ genes/g of sediment. As such, SRB abundance decreases with depth, with one-way ANOVA confirming that the abundance in the surface sediment is significantly different from the abundance in the two deeper layers.

## Discussion

Pore-water sulphate concentration decreases from 14.9 to 3.6 mM in the top centimeters and remains low in the deeper sediment, indicating a near-surface sulphate reduction zone, as observed elsewhere [24-29]. Sulphate concentration in seawater and marine sediments is around 28 mM [11]. Mangroves are brackish ecosystems, due to tidal activity, and have a higher sulphate concentration than freshwater sediments.

In accordance with the sulphate profile, q-PCR showed a significantly larger population of *dsr*-containing microorganisms in the 0–5 cm layer relative to the deeper sediments. This is consistent with the sulphate-reduction zone being located in the shallower sediment interval and suggests that SRB populations are active there. High microbial abundance in the shallow sulphate-containing sediment was also reported in previous studies [28], where it was associated with intense sulphate reducing activity likely owing to organic matter availability.

DGGE was used to assess the sediment bacterial community, using as targets the genes encoding 16S rRNA, BamA and DsrAB. DGGE analysis of 16S rRNA gene diversity revealed depth-dependent differences. A distinct bacterial community composition was identified below 5 cm (i.e., below the sulphate-reduction zone) and is similar in the two deeper sediments, possibly due to lower organic matter availability.

Positive PCR amplification of *bamA* indicates the potential for anaerobic aromatic hydrocarbon-degrading microorganisms at all sediment depths. BamA is involved in the degradation of aromatic hydrocarbons in general, not only petroleum-derived aromatics. BamA-encoding microorganisms are found in the environment independently of contamination [20,30]. Plant matter is a major source of aromatic hydrocarbons [31], which may explain the prevalence of BamA-encoding microorganisms throughout the sediment. Alternatively spilled crude oil percolates deep into the sediment, and the close contact with aromatic compounds in more recalcitrant crude oil fractions might enrich *bamA* containing microorganisms. The apparent absence of Bss-encoding bacteria might be explained because the *bssA* variants targeted by our PCR primers may be mainly involved in anaerobic degradation volatile aromatic compounds (e.g., toluene and o-xylene [22]) which evaporate soon after the oil is spilled. Alternatively, other metabolic pathways and functional genes could be involved in the degradation of oil-derived aromatics in this mangrove sediment. The DGGE analysis of *bamA* diversity showed that the population structure of aromatic hydrocarbon degrading bacteria also changes with depth, being more similar in the top 20 cm, where the influence of plant detritus is greatest. In deeper sediment, 35–40 cm, the DGGE pattern contains fewer bands than the other two analyzed depths.

Küntze and colleagues [20] recommended the combination of PCR for *bamA*, which gives an overview of the anaerobic aromatic hydrocarbons degrading microorganisms present in the studied material, with PCR for *bssA*, which is specific for toluene and xylene degradation – although this gene also seems to be involved in the degradation of some long-chain aromatic hydrocarbons (L. Andrade, unpublished data). In the current study, sediment samples from the three depths tested negative for *bssA* (data not shown). Samples were also similarly screened with PCR primers targeting *assA*, involved in anaerobic alkane degradation, and results were also negative. Our failure to amplify *bssA* and *assA* do not necessarily mean that anaerobic aromatic hydrocarbon-degrading microorganisms are absent from the Surui mangrove sediment; they may be present at abundances too low to be detected with the PCR protocol used. Alternatively, anaerobic hydrocarbon degraders possessing *ass*/*bss* sequence variants lacking homology to our PCR primers [18] or that employ degradation pathways altogether different to the ones tested here (e.g., carboxylation reactions [32] or the two-step oxidation of methylene observed in the degradation of ethylbenzene by a nitrate-reducing strain [33]) for catabolism of anaerobic hydrocarbons.

PCR-DGGE analyses for *dsr* showed that the bacterial community profile in the top 5 cm differs from the two deeper sediment intervals, which was also observed in DGGE analysis of 16S rRNA genes. Nevertheless, the similarities in banding pattern are large concerning sediments of the two deeper layers, while both change a little when comparing to superficial sediment. Similar diversity among dissimilatory sulfite reductase sequences in deeper sediment layers was also observed by Fan and colleagues [34] who analysed *dsrAB* from the surface to 50 cm depth. They suggest that different surficial and deeper sediment SRB community structure is related to tidal variation, which makes sediment temporarily oxic, hypoxic or anoxic. Moreover, tidal inundation also transports sulphate from the sea to the coastal sediment, which shows a high sulphate concentration in the first centimetres of sediment, but diluted in the freshwater presents a low concentration downward. Taketani and colleagues [35] also studied SRB community structure using DGGE and showed that SRB diversity decreases with depth in mangrove sediment, as well as revealing a drop in the relative abundance of SRB, in agreement with the qPCR results presented here (Figure 4). However they noted little variation in diversity in the first 30 cm of that sediment [35]. In anaerobic sediments, SRB play an important role in nutrient cycling and organic matter remineralization [6], and they can be especially important in oil-polluted locations where certain SRB are capable of anaerobic hydrocarbon degradation [11]. Taketani and colleagues confirmed the importance of SRB populations in mangrove sediments, particularly after an oil-contamination event. In a study using mesocosms with pristine and polluted mangrove sediments, they reported an increase in SRB abundance in pristine sediment after oil input, and observed that a mangrove with history of oil contamination is better prepared to respond to such an adverse situation than a non-contaminated one [7].

General bacterial abundance determined by 16S rRNA-targeted qPCR was highest in the 0–5 cm layer sediment, and decreased with depth (Figure 4). The same phenomenon occurs for sulphate-reducing bacteria, in agreement with sulphate concentrations measured in the sediment depths investigated. Comparing q-PCR results for *dsr* and 16S rRNA gene fragment genes suggests that a large fraction of the bacteria present may be sulphate-reducers. It is remarkable that in the top sediment, *dsr* genes represent almost 80% of the number of genes for general bacteria (16S rRNA gene encoding fragment gene). For the deeper sediments these values are almost 40% (15–20 cm) and almost 65% (35–40 cm). It is well known that microorganisms contain more than one copy of 16S rRNA gene. This also might happen for *dsr* gene [36]. Moreover, the primers for 16S rRNA gene encoding fragment gene used in the present study target bacteria, while in their study, Geets and colleagues [36] also detected archaeal *dsr* with the same primer pair that was used here. In principle *dsr* detected in these mangrove sediments by q-PCR could have archaeal species, and as such, the values we report could overestimate the number of sulphate-reducing bacteria.

This is one of the few studies on anaerobic bacterial diversity in mangrove sediments at different sediment depths. Results presented in this study shows that the bacterial diversity and abundance change with depth. This might explain why petroleum and other xenobiotic compounds that percolate to the deep anoxic sediment layers may remain undegraded for years.

## Conclusions

Sulphate decreases dramatically in the first centimetres of the mangrove sediment, and overall bacterial diversity and abundance from the surficial interval (0–5 cm) differs from deeper layers (15–20 and 35–40 cm), which are very similar to each other. Genes involved in anaerobic alkane and aromatic petroleum hydrocarbon degradation were not detected by PCR, perhaps because gene targets for the PCR primers chosen may not have matched to *in situ* genetic diversity.

## Methods

### Sediment sampling

**Figure 5:**
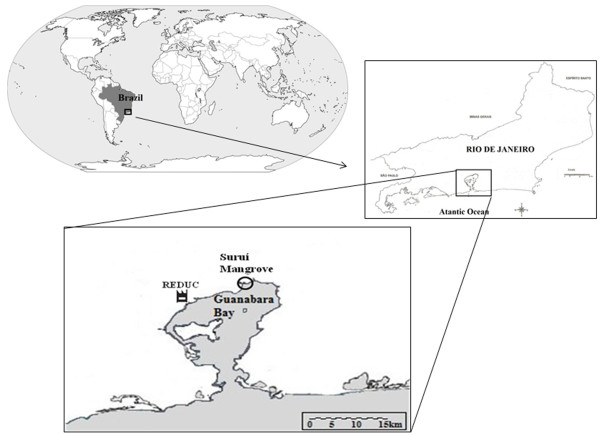
**Suruí Mangrove location.** Location of the Suruí Mangrove. The oil refinery nearby is indicated by .

The sampling site was the Suruí mangrove in Guanabara Bay, situated in Magé, state of Rio de Janeiro, Brazil (Figure 5). In the year 2000, there was an oil spill in Guanabara Bay, impacting the Suruí mangrove. More than 1 million liters of oil leaked from a broken pipeline of an oil refinery nearby, and the most affected region was the northern part of the bay [37].

Samples were collected at one point of the mangrove (S 22º41’50”, W 043º07’00”), during the low tide period. Four aluminum tubes 60 cm in length were used to obtain sediment cores down to 40 cm depth, with less than 1 m of distance of each other sampling point. After sampling, tubes were wrapped in plastic material to limit oxygen exposure, and transported immediately to the laboratory for further processing steps.

In the laboratory, each core was sectioned to obtain samples of the following intervals: 0–5, 15–20 and 35–40 cm deep. Sediment samples of the four replicate cores for each interval were each divided into two parts: a portion reserved for total genomic DNA extraction and molecular based studies, and another one reserved for porewater sulphate analysis.

### Sediment porewater sulphate concentration

Sulphate was analysed by chromatography through Metrohm ion chromatograph with conductivity detection, isolated in a 100 × 4.0 mm polyvinyl ethanol column, using sodium carbonate and sodium bicarbonate as eluent.

### Molecular techniques for sediment: PCR-DGGE for 16S rRNA, *bamA* and *dsr* genes

Total genomic DNA was extracted from bulk sediment of each replicate using FastDNA® SPIN kit, accordingly to manufacturer recommendations.

PCR reactions for further DGGE analysis were performed using U968f-GC1 and L1401, universal primers for the 16S rRNA gene, as previously described by Heuer and Smalla [38]. Before DGGE analysis, PCR products were confirmed to have been amplified by electrophoresis in a 1.2% agarose gel run at 80 V in Tris-Borate-EDTA buffer, and further staining step for 15 min immerse in a solution containing 0.5 g/ml ethidium bromide and revealed under short-wavelength ultraviolet light.

PCR products were submitted to DGGE analysis [39] using a DCode System (universal mutation detection system, BioRad, Richmond, USA), using a 6% acrylamide gel within a denaturing gradient of 40% to 70% of a mixture of urea and formamide. Electrophoresis was performed in 1x Tris-acetate-EDTA buffer at 60°C and at 75 V for 16 h. For the staining step, Sybr Gold (Invitogen) was used, and the gel was visualised using a Storm 860 Imaging System (GE Healthcare). DGGE images were analysed using BioNumerics software (Applied Maths, Belgium) and similarities between lanes were calculated using the band-based Jaccard correlation coefficients, and cluster analysis was performed by the unweighted pair group method with average linkages (UPGMA). PCR-DGGE was also performed for *bamA* to compare the profile of diversity of anaerobic hydrocarbon-degrading bacteria at the three studied depths. PCR mixture and conditions for the *bamA* reactions were as previously described by Küntze and colleagues [20]. Primers SP9 and ASP1 were used and PCR products run on a 9% acrylamide gel within a denaturing gradient of 50% to 70% of urea and formamide. To primer SP9 a GC clamp (5’-CGC CCG GGG CGC GCC CCG GGC GGG GCG GGG GCA CGG GGG G-3’) was attached to stabilize transition forms of the DNA molecule, improving band pattern results [40].

To compare the diversity of SRB at different depths, a PCR-DGGE was executed using two pairs of primers for *dsr* gene (Table 1). Formerly, a PCR reaction was carried out using the Primer Set 1. The resulting amplicons of this reaction became templates for a second PCR reaction using Primer Set 2.

**Table 1:** Primers for sulphate-reducing bacteria detection

	**Primer Set**	**Forward (F) and Reverse (R) Oligonucleotide Primer Sequences**	**Reference**
Primer Set 1	DSR1F	F: 5’-ACS CAC TGG AAG CAC GGC GG-3’	[23]
	DSR4R	R: 5’-GTG TAG CAG TTA CCG CA-3’	[36]
Primer Set 2	DSRp2060F-GC	F: 5’-CGC CCG CCG CGC CCC GCG CCC GGC CCG CCG CCC CCG CCC CCA ACA TCG TYC AYA CCC AGG G-3’	[36]
	DSR4R	R: 5’-GTG TAG CAG TTA CCG CA-3’	[36]

Oligonucleotide primers used in PCR reactions for assessment of the sulphate-reducing bacterial communities and comparison between the 3 studied depths.

Reaction with Primer Set 1 consisted of a 25 μl mixture, containing 1× 100 mM Tris–HCl (pH 8.8 at 25°C), 500 mM KCl, 0.8% (v/v) Nonidet P40 (Fermentas), 1.75 mM MgCl_2_, 50 mM of each dNTP, 200 nM of each oligonucleotide primer (Set 1), 2.5 U of Taq DNA polymerase (Fermentas), 0.5 μl of bovine serum albumin (BSA) 1% (V/V), and 1 μl of DNA. Amplification conditions comprised an initial denaturation step of 94°C for 5 min, followed by 30 cycles of 94°C for 30 s, 55°C for 30 s and 72°C for 90 s, and a final extension step of 72°C for 10 min. PCR with Primer Set 2 consisted of a 50 μl mixture, containing 1x 100 mM Tris–HCl (pH 8.8 at 25°C), 500 mM KCl, 0.8% (v/v) Nonidet P40 (Fermentas), 1.75 mM MgCl_2_, 50 mM of each dNTP, 200 mM of each oligonucleotide primer (Set 2), 2.5 U of Taq DNA polymerase (Fermentas), 0.5 μl of bovine serum albumin (BSA) 1% (v/v), and 2 μl of amplicon from the previous reaction. Amplification conditions comprehended an initial denaturation step of 95°C for 5 min, followed by 20 cycles of 95°C for 40 s, 65 down to 55°C (−0.5°C at each cycle) for 1 min and 72°C for 1 min, 20 cycles of 94°C for 40 s, 55°C for 40 s and 72°C for 1 min, and a final extension step of 72°C for 5 min. Amplification success was confirmed with electrophoresis on agarose gel 1.2% (m/v) in TBE buffer 0.5x at 90 V for 90 min. Gel was stained in a solution of GelRedT™ 1x (Biotium, CA, USA). PCR products of the second reaction were separated based on GC composition by DGGE analysis, using 9% acrylamide gel within a denaturing gradient of 45% to 65% of urea and formamide.

### Molecular techniques for bulk sediment: PCR for *assA* and *bssA*

To assess the presence of potential anaerobic hydrocarbon degraders at the mangrove, bulk sediment of the three studied depths were submitted to PCR targeting the genes responsible for anaerobic alkane degradation, and anaerobic toluene and xylene degradation. For these the oligonucleotide primers used were *assA 2 F/R* (Aitken *et al.*, unpublished observations) and *bssA*[22] (Table 2). PCR mixture for *assA 2 F/R* consisted of a 50 μl mixture, containing 5 μl of 10x buffer, 1.5 mM MgCl_2_, 0.2 mM dNTP, 0.2 μM of each primer, and 1 U of Taq DNA polymerase (Promega), and 1 μl of the template DNA. Amplification conditions included an initial denaturation step of 95°C for 5 min, followed by 35 cycles of 94°C for 1 min, 56°C for 1 min and 72°C for 1 min, and a final extension step of 72°C for 5 min. PCR mixture and conditions for *bssA* followed what was previously described elsewhere [23].

**Table 2:** Primers for anaerobic hydrocarbon degradation genes detection

**Primer Set**	**Forward (F) and Reverse (R) Oligonucleotide Primer Sequences**	**Expected amplicon size (bp)**	**Reference**
SP9/ASP1 (*bamA*)	F: 5`-CAG TAC AAY TCC TAC ACV ACB G-3`	~300	[20]
	R: 5`-C MAT GCC GAT YTC CTG RC-3`		
*assA2F/R* (*assA*)	F: 5’-YAT GWA CTG GCA CGG MCA-3’	440	Aitken *et al.*, unpublished observations
	R: 5’-GCR TTT TCM ACC CAK GTA-3’		
7772 F/8546R (*bssA*)	F: 5’-GAC ATG ACC GAC GCS ATY CT-3’	~794	[22]
	R: 5’-TCG TCG TCR TTG CCC CAY TT-3’		

Oligonucleotide primers used in PCR reactions for anaerobic hydrocarbon degradation detection.

### Molecular techniques for bulk sediment: q-PCR for 16S rRNA and *dsr* genes

Quantitative PCR (q-PCR) assays were carried out using ABIPrism 7500 (Applied Biosystems) detection system, to quantify abundance of the gene encoding the 16S rRNA, following manufacturer’s recommendations.

Amplification consisted of a 25 μl reaction containing 12.5 μl of GoTaq® q-PCR Master Mix 2x (Promega), 40 mM Tris–HCl (pH 8.4), 100 mM KCl. 6 mM MgCl_2_, 400 μM dATP, 400 μM dCTP, 400 μM dGTP, 800 μM dUTP, 40 U/ml UDG (Invitrogen), 200 nM of each primer, 0.5 μl ROX Reference Dye 50 mM (Invitrogen), 0.5 μl BSA (1 mg/ml), 5.5 μl H_2_O and 2 ng DNA. Oligonucleotide primers used were 357 F (5’-CTA CGG GRS GCA G-3’) and 529R (5’-CGC GGC TGC TGG CAG-3’), modified from Muyzer and colleagues [39]. The assays were performed in triplicates. A standard DNA sample was previously used to make a standard curve, and H_2_O was used as the negative control. PCR conditions consisted of an initial denaturation step of 94°C for 3 min, followed by 30–40 cycles of 95°C for 1 min, 55°C for 1 min and 72°C for 45 s.

A q-PCR was also used to quantify SRB population, with ABIPrism 7500 (Applied Biosystems) detection system, to quantify abundance of the gene *dsr*. Amplification step was carried out with a 25 μl mixture containing 12.5 μl of GoTaq® q-PCR Master Mix 2x (Promega), 0.5 μl of each primer 10 μM, 0.5 μl BSA (1 mg/ml), 4.5 μl H_2_O and 2 ng DNA [41]. Oligonucleotide primers used were DSR1F (5’-ACS CAC TGG AAG CAC GGC GG-3’) and DSR-R (5’-GTG GMR CCG TGC AKR TTG G-3’) [23]. PCR conditions consisted of an initial denaturation step of 95°C for 5 min, 35 cycles of 95°C for 1 min, 57°C for 1 min and 72°C for 45 s. All samples were used in triplicates and H_2_O was used as the negative control.

To both reactions (16S rRNA and *dsr* gene) efficiencies and melting curves were determined and analysed using ABIPrism 7500 Detection System (Applied Biosystems).

## Abbreviations

BSA: Bovine serum albumin; DGGE: Denaturing gradient gel electrophoresis; PCR: Polymerase chain reaction; PRAS: Pre-reduced anaerobic sterilized; q-PCR: Quantitative polymerase chain reaction; SRB: Sulphate reducing bacteria; UPGMA: Unweighted pair group method with average.

## Authors’ contributions

LLA conceived of the study, and participated in its design and coordination and wrote the manuscript. DCAL carried out some of the molecular genetic studies. EMF helped with sampling and processing steps. LQF and GRP helped with anaerobic manipulation of samples and design of the experiments. MJM participated in the data interpretation. CH participated in the data interpretation and writing. RSP helped in the experiment design, data interpretation and wrote the manuscript. RMCPD and ASR were the major responsible by the experiment design, and helped in data interpretation and wrote the manuscript. All authors read and approved the final manuscript.

## References

[B1] Merhi ZO Gulf Coast oil disaster: impact on human reproduction Fertil Steril 2010 94 1575 1577 10.1016/j.fertnstert.2010.08.036 20828685

[B2] Mitsch WJ The 2010 oil spill in the Gulf of Mexico: What would Mother Nature do? Ecological Engineering 2010 36 1607 1610 10.1016/j.ecoleng.2010.08.009

[B3] Head IM Jones DM Röling WFM Marine microorganisms make a meal of oil Nat Rev Microbiol 2006 4 173 182 10.1038/nrmicro1348 16489346

[B4] Olguín EJ Hernández ME Sánchez-Galván G Contaminación de manglares por hidrocarburos y estratégias de biorremediación, fitorremediación y restauración Ver Int Contam Ambient 2007 23 139 154

[B5] Dias ACF Andreote FD Rigonato J Fiore MF Melo IS Araújo WL The bacterial diversity in a Brazilian non-disturbed mangrove sediment Antonie van Leeuwenhoeck 2010 98 541 551 10.1007/s10482-010-9471-z 20563848

[B6] Lyimo TJ Pol A Harhangi HR Jetten MSM den Camp HJMO Anaerobic oxidation of dimethylsul¢de andmethanethiol in mangrove sediments is dominated by sulfate-reducing bacteria FEMS Microbiol Ecol 2009 70 483 492 10.1111/j.1574-6941.2009.00765.x 19744237

[B7] Taketani RG Franco NO Rosado AS van Elsas JD Microbial community response to a simulated hydrocarbon spill in mangrove sediments J Microbiol 2010 48 7 15 10.1007/s12275-009-0147-1 20221723

[B8] Li C-H Zhou H-W Wong Y-S Tam NF-Y Vertical distribution and anaerobic biodegradation of polycyclic aromatic hydrocarbons in mangrove sediments in Hong Kong, South China Sci Total Environ 2009 407 5772 5779 10.1016/j.scitotenv.2009.07.034 19683792

[B9] Burns KA Codi S Contrasting impacts of localised versus catastrophic oil spills in mangrove sediments Mangroves and Salt Marshes 1998 2 63 74 10.1023/A:1009959529039

[B10] Ke L Yu KSH Wong YS Tam NFY Spatial and vertical distribution of polycyclic aromatic hydrocarbons in mangrove sediments Sci Total Environ 2005 340 177 187 10.1016/j.scitotenv.2004.08.015 15752500

[B11] Widdel F Knittel K Galushko A Timmis KN Anaerobic hydrocarbon-degrading microorganisms: an overview Handbook of hydrocarbon and lipid microbiology 2010 Germany: Springer-Verlag Berlin Heidelberg 1997 2022

[B12] Boopathy R Anaerobic degradation of No. 2 diesel fuel in the wetland sediments of Barataria-Terrebonne estuary under various electron acceptor conditions Biores Technol 2003 86 171 175 10.1016/S0960-8524(02)00162-1 12653283

[B13] Boopathy R Anaerobic biodegradation of no. 2 diesel fuel in soil: a soil comumn study Biores Technol 2004 94 143 151 10.1016/j.biortech.2003.12.006 15158506

[B14] Boopathy R Shields S Nunna S Biodegradation of crude oil from the BP oil spill in the marsh sediments of Southeast Louisiana, USA Appl Biochem Biotechnol 2012 10.1007/s12010-012-9603-1 22350940

[B15] Rabus R Jarling R Lahme S Kühner S Heider J Widdel F Wilkes H Co-metabolic conversion of toluene in anaerobic n-alkane degrading bacteria Environ Microbiol 2011 13 2576 2586 10.1111/j.1462-2920.2011.02529.x 21880102

[B16] Grossi V Cravo-Laureau C Guyoneaud R Ranchou-Peyruse A Hirschler-Réa A Metabolism of n-alkanes by anaerobic bacteria: a summary Org Geochem 2008 39 1197 1203 10.1016/j.orggeochem.2008.02.010

[B17] Callaghan AV Warwik B Chadain SMN Young LY Zylstra GJ Anaerobic alkane-degrading strain AK-01 contains two alkylsuccinate synthase genes Biochem Bioph Res Commun 2008 366 142 148 10.1016/j.bbrc.2007.11.094 18053803

[B18] Callaghan AV Davidova IA Savage-Ashlock K Parisi VA Gieg LM Suflita JM Kukor JJ Wawrik B Diversity of benyzl- and alkylsuccinate synthase genes in hydrocarbon-impacted environments and enrichment cultures Environ Sci Technol 2010 44 7287 7294 10.1021/es1002023 20504044

[B19] Heider J Fuchs G Anaerobic metabolism of aromatic compounds Eur J Biochem 1997 243 577 596 10.1111/j.1432-1033.1997.00577.x 9057820

[B20] Küntze K Shinoda Y Moutakki H McInerney MJ Vogt C Richnow H Boll M 6-Oxocyclohex-1-ene-1-carbonyl-coenzyme A hydrolases from obligately anaerobic bacteria: characterization and identification of its gene as a functional marker for aromatic compounds degrading anaerobes Environ Microbiol 2008 10 1547 1556 10.1111/j.1462-2920.2008.01570.x 18312395

[B21] Beller HR Kane SR Legler TC Alvarez PJJ A real-time polymerase chain reaction method for monitoring anaerobic hydrocarbon-degrading bacteria based on a catabolic gene Environ Sci Technol 2002 32 3977 3984 12269751 10.1021/es025556w

[B22] Winderl C Schaefer S Lueders T Detection of anaerobic toluene and hydrocarbon degraders in contaminated aquifers using benzylsuccinate synthase (*bssA*) genes as a functional marker Environ Microbiol 2007 9 1035 1046 10.1111/j.1462-2920.2006.01230.x 17359274

[B23] Kondo J Nedwell DB Purdy KJ Silva SQ Detection and enumeration of sulphate-reducing bacteria in estuarine sediments by competitive PCR Geomicrobiol J 2004 21 145 157 10.1080/01490450490275307

[B24] Macdonald BCT Smith J Keene AF Tunks M Kinsela A White I Impacts of runoff from sulfuric soils on sediment chemistry in an estuarine lake Sci Total Environ 2004 329 115 130 10.1016/j.scitotenv.2004.02.016 15262162

[B25] Leloup J Loy A Knab NJ Borowski C Wagner M Jørgensen BB Diversity and abundance of sulfate-reducing microorganisms in the sulfate and methane zones of a marine sediment, Black Sea Environ Microbiol 2007 9 131 142 10.1111/j.1462-2920.2006.01122.x 17227418

[B26] Leloup J Fossing H Kohls K Holmkvist L Borowski C Jørgensen BB Jørgensen BB Sulfate-reducing bacteria in marine sediment (Aarhus Bay, Denmark): abundance and diversity related to geochemical zonation Environ Microbiol 2009 11 1278 1291 10.1111/j.1462-2920.2008.01855.x 19220398

[B27] Habicht KS Gade M Tharndrup B Berg P Canfield DE Calibration of sulphate levels in the Archean Ocean Science 2002 298 2372 2374 10.1126/science.1078265 12493910

[B28] Chatterjee S Dickens GR Bhatnagar G Chapman WG Dugan B Snyder GT Hirasaki GJ Pore water sulfate, alkalinity, and carbon isotopes profiles in shallow sediment above marine gas hydrate systems: a numerical modelling perspective J Geophys Res 2011 116 B09103 10.1029/2011JB008290

[B29] Lyimo TJ Pol A den Camp HJMO Sulfate reduction and methanogenesis in sediments of Mtoni mangrove forest, Tanzania Ambi 2002 31 614 616 12572833

[B30] Staats M Braster M Röling WFM Molecular diversity and distribution of aromatic hydrocarbon-degrading anaerobes across a landfill leachate plume Environ Microbiol 2011 13 1216 1227 10.1111/j.1462-2920.2010.02421.x 21281422

[B31] Lahme S Eberlein C Jarling R Kube M Boll M Wilkes H Reinhardt R Rabus R Anaerobic degradation of 4-methylbenzoate via a specific 4-methylbenzoyl-CoA pathway Environ Microbiol 2012 14 1118 1132 10.1111/j.1462-2920.2011.02693.x 22264224

[B32] Spormann AM Widdel F Metabolism of alkylbenzenes, alkanes, and other hydrocarbons in anaerobic bacteria Biodegradation 2000 11 85 105 10.1023/A:1011122631799 11440245

[B33] Rabus R Heider J Initial reactions of anaerobic metabolism of alkylbenzenes in denitrifying and sulfate-reducing bacteria Arch Microbiol 1998 170 377 384 10.1007/s002030050656

[B34] Fan L-F Tang S-L Chen C-P Hsieh H-L Diversity and composition of sulfate- and sulfite-reducing prokaryotes as affected by marine freshwater gradient and sulfate availability Microbiol Aquatic Sys 2011 63 224 237 10.1007/s00248-011-9912-x 21785985

[B35] Taketani RG Yoshiura CA Dias ACF Andreote FD Tsai SM Diversity and identification of methanogenic archaea and sulphate-reducing bacteria in sediments from a pristine tropical mangrove Antonie van Leeuwenhoeck 2010 97 401 411 10.1007/s10482-010-9422-8 20195901

[B36] Geets J Borremans B Diels L Springael D Vangronsveld J Lelie D Vanbroekhoven K DsrB gene-based DGGE for community and diversity surveys of sulphate-reducing bacteria J Microbiol Methods 2006 66 194 205 10.1016/j.mimet.2005.11.002 16337704

[B37] Michel J Assessment and recommendations for the oil spill cleanup of Guanabara Bay, Brazil Spill Sci Technol Bull 2000 6 89 96 10.1016/S1353-2561(00)00056-6

[B38] Heuer H Smalla K Elsas JD, Trevors J, Wellington EMH Application of denaturing gradient gel electrophoresis (DGGE) and temperature gradient gel electrophoresis for studying soil microbial communities Modern Soil Microbiology 1997 New York: Marcel Dekker 353 373

[B39] Muyzer G De Waal EC Uitterlinden AG Profiling of complex microbial populations by denaturing gradient gel electrophoresis analysis of polymerase chain reaction-amplified genes coding for 16S rRNA Appl Environ Microbiol 1993 59 695 700 7683183 10.1128/aem.59.3.695-700.1993 PMC202176

[B40] Rosado AS Duarte GF Mello IS, Valadares-Inglis MC, Nass LL, Valois ACC Utilização de eletroforese em gel com gradiente de desnaturantes (DGGE) e gel com gradiente de temperatura (TGGE) para estudar a diversidade microbiana Genética e melhoramento de microrganismos 2002 Jaguariúna: Embrapa Meio Ambiente 97 128

[B41] Spence C Whitehead TR Cotta MA Development and comparison of SYBR Green quantitative real-time PCR assays for detection and enumeration of sulfate-reducing bacteria in stored swine manure J Appl Microbiol 2008 105 2143 2152 10.1111/j.1365-2672.2008.03900.x 19120660

